# Clinical profile of children with posterior urethral valve at Tertiary Care Center

**DOI:** 10.12669/pjms.38.7.5823

**Published:** 2022

**Authors:** Rabia Yamin, Khemchand Moorani, Mehmood Shaikh, Sidra Yamin

**Affiliations:** 1 Dr. Rabia Yamin, Resident, National Institute of Child Health, Karachi, Pakistan; 2 Prof. Khemchand N Moorani, Professor of Pediatric Nephrology, The Kidney Center Postgraduate Training Institute (TKC-PGTI), Karachi, Pakistan; 3 Dr. Mehmood Shaikh, Assistant Professor, National Institute of Child Health, Karachi, Pakistan; 4 Sidra Yamin, IPP, Bahria University, Islamabad, Pakistan

**Keywords:** Congenital obstructive uropathy, Boys, Urinary tract infection, Posterior Valve resection

## Abstract

**Background & Objective:**

Posterior Urethral Valves (PUV) are common cause of congenital obstructive uropathy in boys and may be associated with urinary tract infection(UTI) and chronic kidney disease(CKD) if not managed timely. The objective of our study was to determine the clinical profile of children with PUV.

**Methods:**

This is a descriptive case series comprising of 30 children aged 1-5 years, diagnosed and managed as PUV over six months, conducted at the Department of Pediatric Nephrology, National Institute of Child Health, Karachi. Patients were followed for 12 weeks and the outcome was assessed in terms of recovery, UTI, urinary incontinence and CKD. Descriptive statics were used for data analysis.

**Results:**

Thirty cases of PUV were managed during study period. Clinical presentations were poor urinary stream (83%), fever (73%), signs and symptom suggesting UTI (96.6%), pallor (73.3%), acute kidney injury (37%)and urinary retention (13%). UTI was confirmed in 73.3 % and *E.Coli* was the most common pathogen. Ultrasonography showed bilateral hydronephrosis/hydroureter in 80% and micturating cystourethrogram demonstrated vesicoureteral reflux in 86.66% cases. All patients received intravenous hydration (97%), urinary decompression, and antibiotics. Meropenem was the most commonly used. Packed cell transfusion and peritoneal dialysis was done in 73.33% and 13.3% respectively. Cystoscopic valve fulguration was done in 86.66% and vesicostomy in 13.3%. On short-term follow-up, 60% recovered,16.66% experienced UTI and remained incontinent whereas 23.33% had CKD.

**Conclusion:**

Our study showed a high frequency of UTI and AKI. E. coli was most common pathogen. Despite valve fulgration, significant patients had CKD.

## INTRODUCTION

Posterior urethral valves (PUV) is an abnormal development of an accessory urethral membrane in the proximal urethra resulting in obstruction of urine outflow from the bladder.^1^ PUV is common obstructive uropathy in boys contributing to etiology of chronic kidney disease(CKD) in 42.7% of cases 2 The reported incidence of PUV is one in 5000 to 8000 live births.^3^ In a recent study from Pakistan PUV has been reported as 34.3% among 140 children with congenital anomalies of kidneys and urinary tract(CAKUT).^4^ Pathophysiological effects of obstruction at proximal urethra leads to an increase in intravesical pressure, dilatation of ureters and pelvicalyceal system resulting in hydroureter, hydronephrosis and VUR. Severe intrauterine obstruction leads to renal parenchymal damage in the form of renal dysplasia. The effects of VUR, recurrent UTI resulting in renal scarring, all these are associated with high risk of CKD.^5^

The clinical spectrum of PUV depends upon the age of presentation and antenatal fetal ultrasound (US) findings. Patients with PUV may present in their early infancy or in older children. In newborns, it may present with respiratory distress, sepsis, azotemia, poor urinary stream, failure to void in the first 24 hours, palpable bladder, abdominal distension as a result of urinary ascites, or recurrent urinary tract infection, failure to thrive, and the manifestation of CKD in older children.^5^

Diagnosis of PUV may be suspected on antenatal US scan as proximal urethral dilatation, hydronephrosis and oligohydramnios.^3^ However, diagnosis is confirmed postnatally by micturating cystourethrogram (MCUG) which may show the classical posterior urethral dilatation and bladder trabeculation.^1^,^6^ Sometimes PUV is diagnosed after cystoscopy for evaluation of bladder dysfunction and UTI in older children.

Management of PUV is surgical and should be attempted after treating or excluding UTI and assessing renal functions. Urethral valve ablation can be achieved by endoscopic valve resection or electro-cauterization (fulguration).^4^ In some patients, renal function does not improve despite valve ablation due to associated renal dysplasia and may progress to CKD.^7^

However, long term renal outcome depends on multiple factors including the age of presentation, serum creatinine at initial diagnosis, age at intervention, prenatal diagnosis, associated, renal dysplasia, scarring, presence of secondary vesicoureteral reflux (VUR), upper tract obstruction, bladder dysfunction and UTI.^5^,^8^

Several studies have been done on different aspects of PUV.^8^-^11^ Though, there are some studies carried out on Pediatric obstructive uropathy in Pakistan like stone disease, PUV and VUR but there is no prospective study on a clinical profile of PUV.^3^,^4^,^11^ This study may give us current pattern of clinical presentation and short-term treatment outcome of these patients managed at tertiary care center. Therefore, the objective of our study was to determine the clinical profile of children with PUV and short- term outcome.

## METHODS

This descriptive case series was conducted at the Department of Pediatric Nephrology, National Institute of Child Health (NICH), Karachi over a period of six months from July 2019 to December 2019. The ethical clearance was taken from the IERB (28/2019) of NICH and consents were taken from parents. There were 30 boys with PUV who underwent diagnostic workup and management during the study period. Patients aged 1-5 years, who presented with suspicion of PUV with one or more than one manifestations such as poor urinary stream, fever, dysuria, hematuria, retention of urine, pallor, palpable bladder, and signs of deranged renal functions were investigated with urinalysis, urine culture and sensitivity, complete blood counts and radio imaging including US and MCUG. The diagnosis was confirmed on MCUG which showed dilated posterior urethra and trabeculated bladder. All patients underwent either cystoscopic valve fulguration / vesicostomy and the outcome was assessed in follow up at 12 weeks in terms of UTI, urinary incontinence and CKD. Children were excluded if already underwent PUV resection, had associated congenital anomalies like duplex system, prune belly syndrome, and spina bifida. Also, children whose parents did not consent and who left against medical advice or lost follow-up were excluded.

The financial status of parents was divided based on monthly income in Pak rupees(Rs) as a low-income group (Rs ≤ 15000), lower-middle (Rs 15000-25000), and middle-income group (Rs 25001-45000).^12^ It was a convenience sampling technique and due to the rarity of PUV, a sample size of 30 cases was taken. Data were collected from all patients including demographic information, anthropometric measurements, various clinical presentations and outcomes, labs parameters including urine culture, complete blood count (CBC), biochemistry, radio imaging like US kidneys, ureter and bladder(KUB), MCUG, dimercaptosuccinic acid (DMSA) kidney scan, and type of surgical procedure. Data was analyzed on SPSS Version 22. Mean and standard deviations were calculated for the quantitative variables like age, height, weight, and duration of symptoms. Frequencies and percentages were calculated for the qualitative variables like gender, clinical presentation and outcome. Paired “t” test was applied to determine the change from initial eGFR at diagnosis to eGFR at 12 week’s follow up. A p-value of ≤ 0.05 was considered significant.

## RESULTS

We studied thirty boys with a mean age of 2.2 ± 0.80 months with a diagnosis of PUV, worked up, managed, and followed up for about 12 weeks’ duration. The majority of patients (n=22, 73.3%) were below six months of age, between six to 12 months were five (16.6% and 3(10%) were above one year. The mean height and weight were 58.97± 12.8 cm and 4.63 ± 3.26 kg respectively. The mean duration of symptoms was 1.27 ± 1.04 months and the majority (n= 24, 80%) of cases had symptoms of more than one month. The educational status of the family showed that the maternal education level was primary or secondary in 10 and 9 families and 11 (36.7%) were illiterate. Family income status showed that 13 (43.3%) were a low-income group and 16 (53.3%) belonged to a lower-middle group and one (3.3%) was from the middle-income group. The majority of families (n=20, 66.7%) were resident of Karachi whereas only 10 (33.3%) were from rural areas of Sindh.

The clinical profile of patients showed that the majority (83%) had a poor urinary stream. Other common presentations were fever (73%), signs and symptoms of UTI (96.6%), pallor (73.3%), AKI (37%) and palpable bladder (13%).

The baseline laboratory parameter of the study population are shown in Table-I. The CBC showed mean hemoglobin 8.92 ± 2.71G/dl, a mean total leukocyte counts 13.86 ±6.45 10x9/L, mean platelet count 344.83 ± 150.76 10x9/L. The mean serum Na137.50 ±7.12, K 4.4 ± 0.80 and Cl 104.06 ± 5.44 mEq/L. The mean urea and creatinine were 75.17 ± 12.07, 1.7 ± 0.27mg/dl respectively. The mean pre and post eGFR was 25.5 ± 22.2 and 68.7 ± 37.8 ml/min per 1.73 m^2^ respectively.

**Table I:** Biochemical, hematological and urinary findings in children with posterior urethral valves (N=30).

Parameters	Mean	SD
***Hematology***		
Hemoglobin(G/dl)	8.92	2.71
Total leucocyte count(10x9/L)	13.86	6.45
Platelet(10x9/L)	384.80	156.66
***Biochemical***		
Urea(mg/dl)	74.27	63.75
Creatinine(mg/dl)	1.65	1.46
Na(mEq/L)	137.50	7.12
K(mEq/L)	4.48	.80
CL(mEq/L)	104.06	5.44

*Urinalysis*	*N*	*%*

Pyuria (>8 cells/hpf)	26	86
Nitrates positivity	17	56.70
Leucocyte Esterase positivity	16	53.30
***Urine culture***		
Positive	22	73.3
Negative	8	26.6

**Fig.1:**
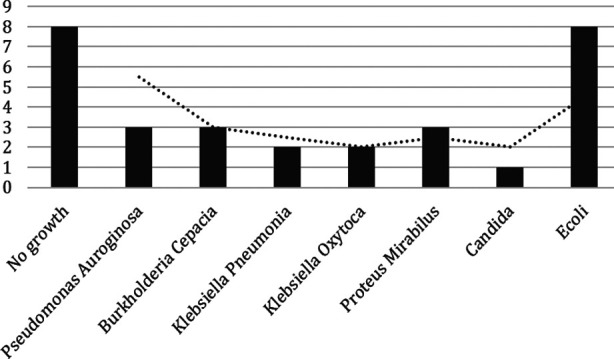
Types of pathogen in children with urinary tract infection.

Urine analysis (Table-I) shows significant pyuria (WBC ≥ 08 /hpf) in 26 and positive nitrites and leukocyte esterase in 17 (56.70%) and 16 (53.3%) respectively. The urine culture was positive in 22 (73.3%) cases. *E.coli* was the most common pathogen (08) followed by Proteus mirabilus, Burkholderia cepacia, Pseudomonas aeruginosa (each in 3 cases), and others (Fig.1).

Ultrasography (Table-II) showed bilateral hydronephrosis and hydroureter in 100% and 80% respectively. Bladder trabeculation and thick-wall were observed in 22 (73%).MCUG showed dilated posterior urethra in 26 (86.33%) patients and VUR either bilateral or unilateral in 28 (93.33%) children. There was high grade VUR (IV= 18, V=15) in majority (60%) cases. Dilated posterior urethra suggestive of PUV was observed in 26(86.33%) cases. DMSA nuclear scan showed that on right side, well-functioning kidneys in 14 (46.7%), fair functioning in 7 (23.3%) and non-functioning in nine (30%) cases while on left side, well- functioning 13(43.3%) fair functioning 8 (26.7%), and non-functioning in seven (23.33%) cases.

**Table II:** Radio imaging characteristics of patients with posterior uretheral valves(n=30).

Ultrasound Findings	Number	Percentage	
***Parameter***			
Hydronephrosis	30	100	
Hydroureter	24	80	
Parenchymal changes	25	83.33	
Dilated Post Urethra	26	86.33	
Bladder trabeculation and wall thickening	22	73.33	

*VUR grading on MCUG(n=26)*	*Right (n=26)*	*Left(n=23)*	*Total*

VUR Grade V	10	5	15
VUR Grade IV	8	10	18
VUR Grade III	5	5	10
VUR Grade I	3	3	6

*DMSA renal Scan Findings(n=30)*	*Right*	*Left*	

Fair -functioning	7	8	
Well-functioning	14	13	
Non-functioning	9	9	

**Fig.2:**
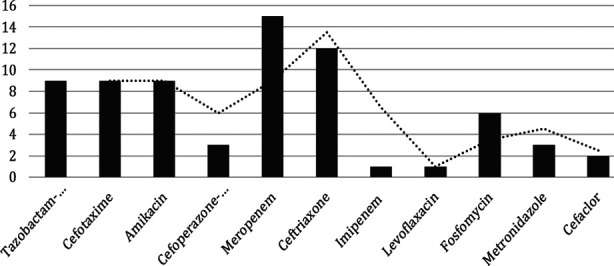
Antimicrobial agents used in children with urinary tract infection.

Patients received intravenous hydration in 96.6% depending upon the clinical status of hydration and urine output. Packed cell transfusion was done in 22 children (73.33%). Initial decompression by catheterization was done in 29 children. Various antimicrobial agents were used in all patients to treat UTI or urosepsis (Fig.2). As per protocol, ceftriaxone or cefoperazone was started before the culture result. Meropenem was the most common antibiotic used in 15 patients followed by ceftriaxone (12), tazobactam, ceftozime, amikacin (each in 9 patients), and fosfomycin (6). Cystoscopic valve fulgration was done in 26 and 4 had temporary vesicostomy (Table III).

**Table III:** Management and outcome of children with posterior urethral valves(n=30).

Management		Yes N (%)	No N (%)
***Parameter***			
Intravenous fluid therapy		29 (96.6)	1 (3.33)
Catherization		29 (96.6)	1 (3.33)
Antimicrobial therapy		30 (100)	-
Peritoneal dialysis		4 (13.3)	26 (86.66)
Vesicostomy		4 (13.3)	26 (86.66)
Valve fulguration		26 (86.66)	4 (13.3)
***Outcome at 3 months***			
Recurrent UTI		5 (16.66)	25 (83.33)
Urinary incontinence		5 (16.66)	25 (83.33)
Improved urinary stream		18 (60)	12 (40)

*Renal Outcome*	*Mean ± SD*	*Mean change*	*p-value*

***eGFR ml/min/1.73m^2^***		

At initial diagnosis At 3 months follow-up	25.5± 22.2 68.7±37.8	-43.2	<0.001
CKD (eGFR <60 ml/min1.73m^2^)			7 (23.33)

At 12 week’s follow-ups, there was an improvement in the urinary stream in 18(60%) and were symptom-free. Five children had urinary incontinence and UTI was observed in five and seven patients had CKD (residual eGFR < 60ml/min /1.73m^2^).

## DISCUSSION

Posterior urethral valves are common obstructive uropathy leading to recurrent UTI, CKD and urinary incontinence in boys. The current study was aimed to determine the clinical profile and short-term outcome of children with the posterior urethral valves at a tertiary care unit of Pakistan.

We observed a mean age of children with PUV as 2.2±0.80 months suggesting the delayed diagnosis, likely to be due to either lack of antenatal US. A high percentage (63.3%) of children were brought to the hospital after the age of one month and 10% after one-year age again highlighting the poor health infrastructure. Similar to our result Nasir et al showed that 41.4% of patients with PUV were brought to the hospital after one year of age.^10^ A similar finding has been reported in a case report who presented as urinary incontinence.^13^ The reasons of delayed presentation in this particular case had been attributed to ignorance, poor health care like our three cases who were brought after one year of age.

Though, 66.7% of our patients were residing in Karachi but presented late suggesting a lack of education among parents (36.7% illiterate mothers) as well as lack of awareness among practicing health personnel for early pickup and referral. Furthermore, financial constraint can be an additional factor behind parents’ reluctance to visit a hospital since the majority belonged to either low 13 (43.3%) or lower-middle-income 16 (53.3%) groups.

The most common presenting complaint in our patients was a poor urinary stream (83%) followed by fever (73%), pallor 22 (73.33%) and palpable bladder (13%). Though poor urinary stream is a classical presentation in these children but pallor indicates either infection or uremia again suggesting late arrival with complication. Dribbling of urine / poor stream has been reported in 51% of children with PUV in other studies.^4^,^9^,^14^ UTI is common in children with distal urinary tract obstruction like PUV. A recent local study has shown UTI in 67 (47.86%) out of 140 children with CAKUT in which 34.3% had PUV. We observed significant pyuria, positive nitrite, and leucocyte esterase in 26 (86%), 17 (56.70%) and 16 (53.30%) cases. Subsequently confirmed by positive bacterial growth in 73.3% of cases and E. Coli as the most common pathogen (36.3%). Similar findings have been reported in our previous study and by others.^15^,^16^ As discussed above more than 70% had culture proven UTI and negative culture in rest of children could be explained by prior use of antibiotic before coming to our center.

Ultrasound showed hydronephrosis and hydroureter in most of the cases suggesting obstructive uropathy. Various grades of VUR were demonstrated by MCUG in our study in 93.33% cases. There was unilateral (n=5) and bilateral(n=10) grade V -VUR. The proportion of secondary VUR in our study is consistent with a recent study by Shakoor J et al and Wanjari M et al.^4^,^17^

Urethral catheterization has been a standard practice to relieve obstruction(decompression) and to improve immediate renal function. We did initial decompression in 97% of cases similar to others.^11^ Temporary diversions like vesicostomy was carried out in 13% due to disparity of size of rescetospoe and urethra. This may allow time to grow the child while preventing infection and further renal damage. Such practice of diversion procedure has been reported by others.^18^,^19^ Endoscopic valve fulguration was done in 27 (93%) of cases. Other studies have reported a similar pattern of endoscopic valve fulguration.^5^,^20^-^22^ The short-term outcome of PUV showed that the urinary stream become normal in 18 (60%) of patients, however episodes of UTI (5, 16.66%) could be explained by residual dilated urinary tract or colonization of the tract with microbial agents.

### Limitations

Major limitations of our study are small number of patients and a short duration of the study period.

## CONCLUSION

Our study showed that majority of children presented with complications of posterior urethral valves like UTI and impaired kidney function. *E. coli* was most common pathogen responsible for UTI. Cystoscopic valve fulgration was successful in 60%. However, CKD was observed in significant number of cases. We recommend study on larger sample and of longer duration of follow-up to find out long term outcome.

### Authors’ contributions:

**KNM:** Conceptualization of idea, guidance in manuscript writing, preparation, final editing and responsible for integrity of study.

**RY:** Data collection, literature search, write up and referencing.

**MS**: Literature search, help in write up and review.

**SY:** Data entry and analysis.
